# Non-invasive vagus nerve stimulation for treatment of cluster headache: early UK clinical experience

**DOI:** 10.1186/s10194-018-0936-1

**Published:** 2018-11-23

**Authors:** Juana Marin, Nicola Giffin, Elizabeth Consiglio, Candace McClure, Eric Liebler, Brendan Davies

**Affiliations:** 1 Wellcome Foundation Building, King’s College Hospital, London, SE5 9PJ UK; 2 Royal United Hospital, Coombe Park, Bath, BA1 3NG UK; 3 Interface Clinical Services, Gate Way Drive, Yeadon, Leeds, LS19 7XY UK; 4 North American Science Associates, Inc., 400 US-169, Minneapolis, MN 55441 USA; 5 electroCore, Inc., 150 Allen Road, Suite 201, Basking Ridge, NJ 07920 USA; 6 University Hospitals of North Midlands, Newcastle Road, Stoke-on-Trent, ST4 6QG UK

**Keywords:** Chronic cluster headache, Refractory, Non-invasive vagus nerve stimulation, Real-world data, Acute treatment, Preventive therapy, Neuromodulation

## Abstract

**Background:**

Evidence supports the use of non-invasive vagus nerve stimulation (nVNS; gammaCore®) as a promising therapeutic option for patients with cluster headache (CH). We conducted this audit of real-world data from patients with CH, the majority of whom were treatment refractory, to explore early UK clinical experience with nVNS used acutely, preventively, or both.

**Methods:**

We retrospectively analysed data from 30 patients with CH (29 chronic, 1 episodic) who submitted individual funding requests for nVNS to the National Health Service. All patients had responded to adjunctive nVNS therapy during an evaluation period (typical duration, 3–6 months). Data collected from patient interviews, treatment diaries, and physician notes were summarised with descriptive statistics. Paired *t* tests were used to examine statistical significance.

**Results:**

The mean (SD) CH attack frequency decreased from 26.6 (17.1) attacks/wk. before initiation of nVNS therapy to 9.5 (11.0) attacks/wk. (*P* < 0.01) afterward. Mean (SD) attack duration decreased from 51.9 (36.7) minutes to 29.4 (28.5) minutes (*P* < 0.01), and mean (SD) attack severity (rated on a 10-point scale) decreased from 7.8 (2.3) to 6.0 (2.6) (*P* < 0.01). Use of abortive treatments also decreased. Favourable changes in the use of preventive medication were also observed. No serious device-related adverse events were reported.

**Conclusions:**

Significant decreases in attack frequency, severity, and duration were observed in these patients with CH who did not respond to or were intolerant of multiple preventive and/or acute treatments. These real-world findings complement evidence from clinical trials demonstrating the efficacy and safety of nVNS in CH.

**Electronic supplementary material:**

The online version of this article (10.1186/s10194-018-0936-1) contains supplementary material, which is available to authorized users.

## Background

Cluster headache (CH), a primary headache disorder, is widely regarded as one of the most painful medical conditions and can substantially diminish patients’ quality of life by limiting their functional abilities in social, domestic, and work activities [1]. The condition may be classified as episodic (attack periods of 1 week to 1 year separated by 1 month) or chronic (attack periods of 1 year without remission or remission of < 1 month) and has limited available treatment options [2, 3]. A non-invasive vagus nerve stimulation (nVNS) device (gammaCore®) has demonstrated safety and efficacy for prevention and acute treatment of CH attacks in clinical trials [4–6]. The device is CE marked and indicated for acute and preventive therapy in CH and for treatment of migraine, hemicrania continua, and medication overuse headache in adults. It is also approved in the United States for acute treatment of episodic CH and migraine in adults.

Understanding the practical role of novel treatments such as nVNS in clinical practice is often difficult, despite clinical trial data demonstrating their efficacy. The use of novel treatments in practice can provide data to complement those from clinical trials by documenting qualitative details that are not typically captured during such trials, enabling a real-world view of patient- and health care–centric management. This can provide a broader view of a treatment’s risk/benefit profile and of patients’ preference and ability to maintain their treatment regimen. To add further insight to the data on nVNS from randomised clinical trials, we conducted this retrospective analysis of data from patients in the United Kingdom with CH who were at various stages in the process of applying for individual funding requests (IFRs) for nVNS from the National Health Service. The IFR process is available to secure financial support for novel therapies that have not been fully evaluated and approved for national reimbursement. The process is reserved for patients with rare conditions that have not responded to available therapies and who are considered exceptional individuals with regard to the treatment of their CH.

## Methods

We retrospectively analysed data from patients with CH who previously had an inadequate response and/or intolerable side effects with ≥3 current or previous CH treatments and were offered nVNS therapy for use during an evaluation period. Physicians instructed patients to use nVNS as preventive therapy, acute treatment, or both during this period. Initial nVNS dosing was based on established paradigms and titrated as necessary to achieve maximum benefit. Patients who reported a clinically meaningful decrease in the frequency, severity, or duration of their attacks after ≥3 months of evaluation were considered for inclusion in the IFR process.

Decreases in the use of concomitant medications and clinical assessments of patient quality of life were also considered. The decision to pursue IFR submission for these subjects was at the discretion of physicians and patients, but submission was not encouraged for patients who did not achieve a ≥ 25% decrease in weekly attack frequency. Patients continued to use nVNS during IFR development, submission, and processing.

All patients provided informed consent for the collection and analysis of their data. Clinical centres provided data on CH attacks and treatments before the nVNS evaluation period, which were obtained from patient diaries and/or medical records, as well as the following data from patient interviews, treatment diaries, and physician notes documented during the nVNS evaluation period (from May 2012 through March 2016): CH type, patient demographics/other characteristics; CH attack frequency, duration, and severity (rated on a 0–10 scale, higher numbers indicating greater severity); number and timing of stimulations administered; concomitant use of preventive and/or abortive treatments; adverse events (AEs); and subjective feedback on nVNS. Data were summarised with descriptive statistics. Within-patient changes from baseline (i.e., during treatment with the standard of care [SoC] regimen alone) to the end (or latest available point) of the nVNS evaluation period in attack frequency, duration, and severity were assessed via paired *t* tests. Patients who were no longer experiencing attacks at the time of the analysis were excluded from analyses of attack duration and severity. Data from patients who lacked quantitative information regarding attack duration and severity were included only in qualitative analysis of these variables.

## Results

### Patient characteristics

Data from 30 patients (Table 1), 29 with chronic CH and 1 with episodic CH, at 10 clinical centres throughout the United Kingdom (see Additional file 1 for list of sites) were analysed.

**Table 1:** Patient demographics and baseline characteristics

Characteristic	SoC + nVNS (*N* = 30)
Age,^a^ mean (range), y	47.9 (16.0–72.0)
Female sex, No. (%)	19 (63)
Diagnosis, No. (%)	
Chronic CH	29 (97)
Episodic CH	1 (3)
Time since CH diagnosis,^a,b^ mean (range), y	7.2 (0–22.0)
Failed preventive treatments,^c^ mean (range), No.	8.9 (1–16)
Failed acute treatments,^c^ mean (range), No.	1.3 (0–4)
Active preventive treatments,^a^ mean (range), No.	0.8 (0–2)
Active acute treatments,^a^ mean (range), No.	1.8 (1–4)

^a^At the time nVNS therapy was begun

^b^Calculated using the year nVNS was begun minus the year CH was diagnosed

^c^Refers to treatments used and stopped before nVNS therapy was begun

Abbreviations: *CH* cluster headache, *nVNS* non-invasive vagus nerve stimulation, *SoC* standard of care

### nVNS use

The mean (range) duration of the evaluation period at the time of analysis was 7.6 (0.9–27.5) months. The most commonly used preventive and acute nVNS regimens are shown in Table 2. Sixteen patients (53%) used nVNS exclusively as preventive therapy, 1 (3%, a patient with episodic CH) used it exclusively as acute treatment, and 13 (43%) used it as both preventive and acute therapy. A single stimulation lasted 120 s, and the mean (range) preventive stimulation frequency was 5.6 (2.0–9.0) stimulations/d. The mean (range) acute stimulation frequency was 4.3 (0.4–18.0) stimulations/d.

**Table 2:** Most commonly used nVNS dosing regimens: preventive and acute treatment

nVNS Dosing Regimen	No. (%)^a^ of Patients
Preventive	
2 consecutive stimulations administered 3 × per day	13 (45)
3 consecutive stimulations administered 2 × per day	8 (28)
Acute	
3 consecutive stimulations administered at the onset of each CH attack	10 (71)

^a^Percentages are based on *n* = 29 patients using nVNS as preventive therapy and *n* = 14 patients using nVNS as acute treatment

Abbreviations: *CH* cluster headache, *nVNS* non-invasive vagus nerve stimulation

### Attack frequency, duration, and severity

**Fig. 1:**
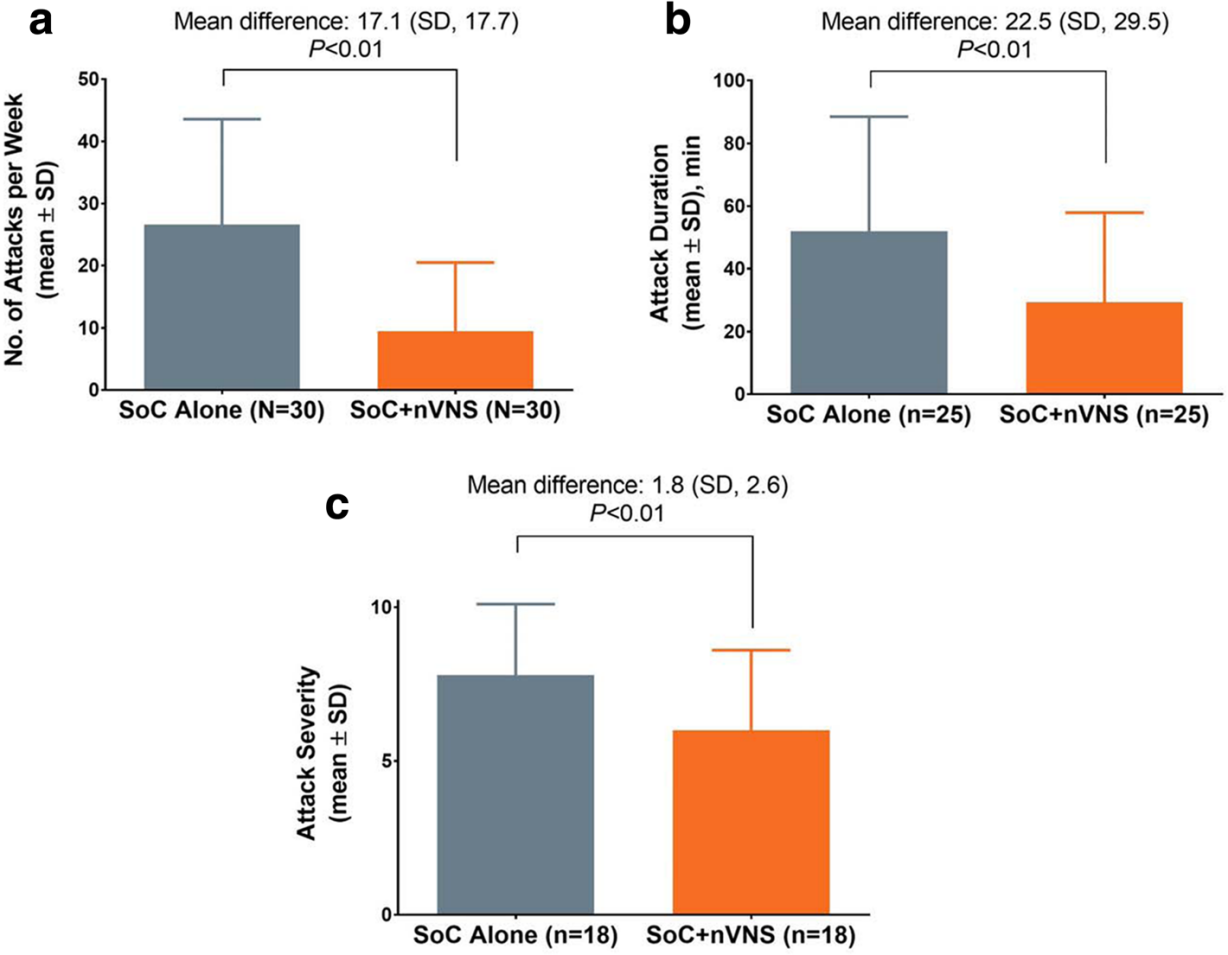
CH attack frequency (**a**), duration (**b**), and severity (**c**) with SoC alone and with SoC + nVNS. *P* values are from paired *t* tests. Patients who had 0 attacks while using nVNS therapy were excluded from analysis of attack duration and severity. Attack severity was rated on a 0 to 10 scale, with higher scores indicating greater severity. Abbreviations: CH, cluster headache; nVNS, non-invasive vagus nerve stimulation; SD, standard deviation; SoC, standard of care

The mean (range) attack frequency with SoC alone was 26.6 (3.8–77.0) attacks/wk.; this decreased to 9.5 (0–38.5) attacks/wk. with SoC + nVNS (*P* < 0.01; Fig. 1a). Three patients, who averaged 42 to 63 attacks/wk. before the initiation of nVNS therapy, had no attacks during their nVNS evaluation periods, which ranged from 1.7 to 13.2 months. Among the 25 patients who reported the duration of their attacks, the mean (range) decreased from 51.9 (5.0–140.0) minutes with SoC alone to 29.4 (2.5–152.5) minutes with SoC + nVNS (*P* < 0.01; Fig. 1b). The mean (range) attack severity (*n* = 18) decreased from 7.8 (3.0–10.0) with SoC alone to 6.0 (1.0–10.0) with SoC + nVNS (*P* < 0.01; Fig. 1c). In the qualitative analysis, most patients reported a decrease in attack frequency, duration, and/or severity during the nVNS evaluation period (Table 3).

**Table 3:** Qualitative analysis of changes in CH attack frequency, duration, and severity with nVNS therapy

	No. (%) of Patients		
	Decrease	No Change	Increase
Frequency	25^a^ (83)	5 (17)	0
Duration^b^	17 (65)	7 (27)	2 (8)
Severity^c^	17 (74)	5 (22)	1 (4)

^a^Included 3 patients who had no CH attacks during the evaluation period

^b^26 patients had available qualitative data on attack duration

^c^23 patients had available qualitative data on attack severity

Abbreviations: *CH* cluster headache, *nVNS* non-invasive vagus nerve stimulation

### Concomitant treatment use

Patients used a mean (range) of 0.8 (0–2) preventive treatments before the initiation of nVNS therapy and 0.7 (0–2) preventive treatments afterward. The mean (range) number of acute treatments used was 1.8 (1–4) before the initiation of nVNS therapy and 1.1 (0–2) afterward. Table 4 summarises the use of acute and preventive medications prior and subsequent to the initiation of nVNS therapy, and Table 5 provides details regarding the use of preventive treatments before the initiation of and concomitant with nVNS therapy. Twenty-two patients used triptan injection or nasal spray as acute treatment before the initiation of nVNS therapy. Among these patients, 9 (41%) stopped and 12 (55%) decreased their triptan use during nVNS therapy; triptan use was unchanged in the remaining patient. Twenty-nine patients reported use of high-flow oxygen; 27 (93%) used it as acute treatment before the initiation of nVNS therapy. After treatment with nVNS was initiated, 9 patients (33%) stopped and 17 (63%) decreased high-flow oxygen use; use of this treatment was unchanged in the remaining patient. Overall, after nVNS therapy was established, 3 patients were able to manage their condition with preventive pharmacologic treatment only, and another 4 patients were able to use nVNS as monotherapy.

**Table 4:** Summary of acute and preventive medication use before and after the establishment of nVNS therapy

	No. of Patients	
	Prior to nVNS	Concomitant With nVNS
Using acute treatment only	14	11
Using preventive treatment only	0	3
Using both acute and preventive treatment	16	12
Using neither acute nor preventive treatment	0	4

Abbreviation: *nVNS* non-invasive vagus nerve stimulation

**Table 5:** Preventive treatment use before and after the establishment of nVNS therapy

Treatment	No. of Patients	
	Prior to nVNS	Concomitant With nVNS
Verapamil	8	6
Topiramate	5	4
Amitriptyline	2	2
Melatonin	2	2
Baclofen	1	1
Bilateral OCS (implanted)	1	1
DBS	1	1
Gabapentin	1	1
Lithium	1	1
Pregabalin	1	0

Abbreviations: *DBS* deep brain stimulation, *nVNS* non-invasive vagus nerve stimulation, *OCS* occipital nerve stimulation

### Safety

No serious device-related AEs were reported during nVNS therapy. Observed AEs in this patient cohort included redness and muscle soreness at the stimulation site, which were also reported in previous randomised clinical trials. Consistent with these previous studies, AEs were mild and transient and were typically reported early in the evaluation period, when the use of nVNS was relatively novel.

### Additional observations and outcomes

In addition to the objective findings, some patients spontaneously reported subjective benefits of nVNS that they considered meaningful. These included decreased interictal headache pain (*n* = 6), no longer being housebound (*n* = 6), the ability to return to work or school (*n* = 4), improved sleep (*n* = 4), decreased absenteeism (*n* = 4), avoidance of surgery intended to treat CH (*n* = 3), and improved quality of life (*n* = 3).

## Discussion

In these patients with CH, headache burden as measured by attack frequency, duration, and severity significantly improved with nVNS therapy. Three patients (10%), all of whom had chronic CH, were attack free after beginning nVNS therapy, which constitutes a remission period according to *International Classification of Headache Disorders* (3rd edition) criteria [3]. Most patients were able to decrease or discontinue their use of existing acute treatments during nVNS therapy.

In previous clinical trials, nVNS demonstrated efficacy as preventive therapy in patients with chronic CH [4] and as acute treatment in patients with episodic CH [5, 6], but not as acute treatment in patients with chronic CH [4–6]. In a recent audit from a single centre in the United Kingdom, data from 12 patients with chronic CH suggested that nVNS was not effective as preventive or abortive therapy for most patients [7]. In contrast, patients in this analysis, who predominantly had chronic CH (29/30), reported significant decreases in attack duration and severity, indicating a benefit from nVNS as an acute treatment in chronic CH in this practical setting when the acute use was added to daily preventive use. These conflicting results suggest that further study is warranted, but the idea of a differential response to nVNS among patients with chronic CH and those with episodic CH is well established and has several possible underlying reasons. There appear to be differences in brain anatomy and pharmacology between patients with episodic CH and those with chronic CH [8, 9]. Disparate changes in grey matter volume during attacks in patients with episodic versus chronic CH, as well as apparent impairment of recovery from such changes between attacks in patients with chronic CH, suggest further differences between the 2 CH subtypes [10]. Suboptimal responses to other acute treatments in patients with chronic CH also have been reported [11, 12]. Results from the initial open-label exploratory study of nVNS therapy in CH suggested that several patients with chronic CH had a stable favourable response to nVNS as acute treatment [13]. In that study, unlike in the aforementioned clinical trials [4–6], nVNS dosing regimens were adjusted according to individual patient responses to explore optimal treatment approaches [13]. In this study, nVNS dosing regimens were also titrated on an individual basis and, importantly, both acute and preventive uses of nVNS were allowed. Such dosing individualisation, which is common with pharmacologic treatments, could explain why patients with chronic CH benefited from acute nVNS treatment in the current study but not in the acute clinical trials, which did not allow for daily preventive use. Further study is needed to determine whether acute treatment regimens in patients with chronic CH might benefit from increased nVNS dosing.

In this report and the initial exploratory study [13], most patients used nVNS as both acute and preventive therapy, which suggests a possible synergy between acute and preventive benefits of nVNS therapy. The possibility that continued or more frequent use of nVNS results in increased efficacy requires further investigation, but some findings suggest this may be the case in CH [6] and migraine [14]. If confirmed, this concept could also help explain the potential synergy between acute and preventive nVNS therapy.

In addition to the clinical benefits of nVNS in CH, an economic benefit of nVNS has also been suggested. Results from a pharmacoeconomic modelling analysis suggested that, compared with SoC alone, SoC + nVNS was associated with 23% lower abortive medication costs and was more effective in patients with CH [15]. Reductions in the use of acute treatments in the current study support the potential cost-effectiveness of nVNS and reiterate its favourable risk/benefit profile.

During clinical trials, patients are instructed to report any AE they experience, regardless of severity, seriousness, or presumed relationship to the study drug/device. In practice, patients are more likely to report only AEs that they find particularly concerning/bothersome or that they believe to be related to treatment. Patients in the current study reported no serious device-related AEs, which provides valuable information regarding how nVNS therapy is tolerated in real-world conditions and helps confirm the mild side effect profile associated with nVNS in clinical trials [4, 5, 14].

The current study sample comprising 63% women is unusual considering that CH is more common among men [16]. Several factors may have contributed to this discrepancy. Compared with men, women with CH have higher rates of comorbidities such as major depression, migraine, and other conditions that could affect the way CH manifests [16, 17]. Such comorbidities might complicate the treatment of CH to the extent that more women than men pursue the IFR process. Concerns about teratogenicity associated with some medications used to treat CH can also prompt women to seek non-pharmacologic treatment options at greater rates than men do. Finally, in our general clinical experience and in this particular patient sample, women are often more willing than men to rigorously and consistently track the data required to complete IFR applications.

Limitations of this study include its small sample size and inherent inclusion bias. By definition, this was a responder study, and patient responses are not likely representative of the CH population as a whole. Use of an evaluation period appears to be a feasible and practical method for assessing response to nVNS in patients with CH, especially if one considers the mild side effect profile of nVNS and practicality of this therapy.

## Conclusions

Treatment with nVNS led to significant decreases in attack frequency, severity, and duration in patients with CH who previously did not benefit from or could not tolerate multiple preventive and/or acute treatments. These findings represent the practical use of this treatment and complement results from clinical trials demonstrating the efficacy and safety of nVNS therapy in patients with CH.

## Additional file

Additional file 1: List of Contributing Study Centres. (DOCX 22 kb)

## Abbreviations

AE: Adverse event
CH: Cluster headache
IFR: Individual funding request
nVNS: Non-invasive vagus nerve stimulation
SD: Standard deviation
SoC: Standard of care
