# Supplementary material for: Non-invasive vagus nerve stimulation for treatment of cluster headache: early UK clinical experience
Source: J Headache Pain. 2018 Nov 23;19(1):114. doi: 10.1186/s10194-018-0936-1 (PMC6755582; doi:10.1186/s10194-018-0936-1)
Supplement: Supplementary file 1 — List of Contributing Study Centres. (DOCX 22 kb) [file 10194_2018_936_MOESM1_ESM.docx]

**Supplemental Material**

**List of Contributing Study Centres(FW regarding sitesconsultants IFR paper)**

Royal Free London NHS Foundation Trust

University College London Hospitals NHS Foundation Trust

The Walton Centre NHS Foundation Trust

The Newcastle upon Tyne Hospitals NHS Foundation Trust

City Hospitals Sunderland NHS Foundation Trust

The Mid Yorkshire Hospitals NHS Trust

University Hospitals of North Midlands NHS Trust

Royal United Hospitals Bath NHS Foundation Trust

King's College Hospital NHS Foundation Trust

Cambridge University Hospitals NHS Foundation Trust
